# Solution scattering study of the *Bacillus subtilis* PgdS enzyme involved in poly-γ-glutamic acids degradation

**DOI:** 10.1371/journal.pone.0195355

**Published:** 2018-04-02

**Authors:** Jumei Zeng, Yun Jin, Zhongchuan Liu

**Affiliations:** Key Laboratory of Environmental and Applied Microbiology, Chengdu Institute of Biology, Chinese Academy of Sciences, Chengdu, Sichuan, China; Università degli Studi di Milano, ITALY

## Abstract

The PgdS enzyme is a poly-γ-glutamic (γ-PGA) hydrolase, which has potential application for a controllable degradation of γ-PGA by enzymatic depolymerization; however, the structure of PgdS is still unknown. Here, to study in detail the full-length PgdS structure, we analyze the low-resolution architecture of PgdS hydrolase from *Bacillus subtilis* in solution using small angle X-ray scattering (SAXS) method. Combining with other methods, like dynamic light scattering and mutagenesis analyses, a model for the full length structure and the possible substrate delivery route of PgdS are proposed. The results will provide useful hints for future investigations into the mechanisms of γ-PGA degradation by the PgdS hydrolase and may provide valuable practical information.

## Instruction

Poly-γ-glutamic acids (γ-PGA) is a water-soluble macromolecular peptide that consists of only D-glutamic acid or D- and L- glutamic acids and is polymerized by γ-glutamyl bonds [1]. γ-PGA is therefore resistant to proteases, which cleave only α-amino bonds. This polymer is synthesized by several bacteria (all Gram-positive) and play different biological roles, like virulence and biofilm formation [2–4]. Because γ-PGA shows water solubility, biodegradation and non-toxicity to human and environment, that makes it widely applicable in many fields, such as food, cosmetics, medicine, chemical industry and so on [5–7].

Several strains of *Bacillus subtilis* and *Bacillus licheniformis* have been widely exploited for producing γ-PGA, due to these organisms produce γ-PGA extracellularly, which simplify recovery and purification of the polymers [8–11]. The PgdS enzyme (also known as YwtD) is a γ-PGA hydrolase from *B*. *subtilis* or *B*. *licheniformis*, which degrades γ-PGA and releases it extracellular into the medium. PgdS is a endo-γ-glutamyl peptidase belonging to the NlpC/P60 family [12], that cleaves only the γ-glutamyl bond between D-glutamic acid and L-glutamic acid of γ-PGA [13]. The PgdS hydrolase exhibits a remarkable activity in γ-PGA degradation over a broad range of temperature (30–40°C) and pH (5.0–8.0) [9]. At the optimal pH and temperature (pH 5.0 and 30°C respectively), an efficient γ-PGA enzymatic degradation is achieved. The molecular weight of γ-PGA can be decreased within the range of 1000–20 kDa and the polydispersity decreases as a function of depolymerization time [9,13]. Therefore, the PgdS hydrolase has potential application for a controllable degradation of γ-PGA by enzymatic depolymerization.

To date, the structure of PgdS is still unknown. Here, we employ a hybrid approach that utilizes small angle X-ray scattering (SAXS) in combination with secondary and tertiary structure prediction to detail the architecture of the PgdS hydrolase from *B*. *subtilis* in solution. Combining with dynamic light scattering and mutagenesis analyses, a model for the structure and the possible substrate delivery route of PgdS are proposed. The results will provide useful hints for future investigations into the mechanisms of γ-PGA degradation by the PgdS hydrolase.

## Materials and methods

### Gene cloning, protein expression and purification

The *pgdS* gene of *B*. *subtilis* 168 (DSM 23778, DSMZ, Germany) were amplified by PCR from genomic DNA with the 5'/3' specific primers. This primer design avoided cloning of the N-terminal signal peptide of 32 residues (predicted by the SignalP 4.1 server [14]). The amplified genes were cloned into vector pGEX-6P-1 and expressed in *Escherichia coli* DH5α with an N-terminal GST-tag. Cells were harvested by centrifugation, re-suspended in lysis buffer and sonicated on ice. Proteins were purified from the supernatant by GST Glutathione SepHaroseTM 4 Fast Flow column (GE Healthcare), and the GST-tag was removed by Prescission Protease (PPase) at 4°C overnight. The eluted PgdS proteins were further purified by the combination of the Resource S anion-exchange column (GE Healthcare) and Superdex 200 size-exclusion column (GE Healthcare) with a final buffer consisting of 50 mM MES (pH 6.0) and 100 mM NaCl. Protein samples were then exchanged into a buffer containing 50 mM citric acid-sodium citrate (pH 5.0) and 100 mM NaCl or 50 mM Tris (pH 8.0) and 100 mM NaCl using centrifugal filters (Amicon Ultracel, EMD Millipore) for the subsequent experiments.

All mutant PgdS proteins were generated according to the QuickChange mutagenesis protocol. All clones were verified by DNA sequencing. These mutants were purified in the same way as described above for the wild type protein.

### SAXS measurements and data processing

Synchrotron SAXS measurements from solutions of PgdS were performed on the BL19U2 beamline at NCPSS (Shanghai, China), equipped with a robotic sample changer and a PILATUS 1M detector [15]. All samples were centrifuged at the speed of 13,000 rpm for 10 min just before measurements to get rid of aggregations and sediments. 2 mM DTT was added into the samples and buffers before measurement to avoid radiation damage. The exposure time of one frame is one seconds. Twenty successive frames were collected for one sample in order to monitor the possible radiation damage. The scattering intensity I(s) was recorded in the range of the momentum transfer, 0.02 < s < 0.4 Å where s = (4πsinθ) / λ, 2θ is the scattering angle, and λ = 1.54 Å is the X-ray wavelength. Because of the high experimental noise for s values > 0.3 Å, the most informative part of the scattering data from 0.02 to 0.3 Å was used for structural analyses. To exclude concentration dependence, different concentrations ranging between 1.1 and 7.2 mg/ml of each sample were prepared and measured. No concentration dependence and aggregations were observed during the measurements. The low angle data collected at lower concentration was merged with the highest concentration high angle data to yield the final composite scattering curve.

All SAXS data were processed with the program package ATSAS [16]. The scattering of buffers were subtracted from that of the samples, and then were extrapolated to zero concentrations using standard procedures and program PRIMUS [17]. The resultant curves were used for all calculations and reconstructions. Low resolution shapes of PgdS were reconstructed by the *ab initio* method, DAMMIF [18]. Twenty models obtained from the program runs were compared and averaged using the program DAMAVER [19], with the most universal model was chosen as typical model. Currently, the high resolution X-ray structure of PgdS from *B*. *subtilis* has not been determined, so a combination of secondary and tertiary structure modeling programs were applied to develop an atomistic representation of PgdS subunits, then the program SASREF [20] was used to determine the relative positions of the subunits. The program CORAL [21] was used to reconstruct missing fragments of the available high-resolution structures using the full amino acid sequences. Considering the flexibility of proteins, program EOM [22] was also used to analyze the PgdS enzyme with assemblies of different conformers.

### Homology structural modeling of the PgdS domains

The 2D secondary structure prediction of PgdS was performed using PsiPred server [23], and the 3D model was generated by SWISS-MODEL[24]. The structure validation and quality control was done by Procheck [25] and WhatCheck module on WhatIf server [26].

### Enzyme assay

PgdS activity was assayed using γ-PGA as the substrate. γ-PGA were reagent grade and purchased from Sigma-Aldrich. γ-PGA (100ug) was incubated with 2 μM enzyme in a 100 μl reaction volume and citric acid-sodium citrate 50 mM, pH 6.0. Reactions were incubated at 37°C for 2 hours, and then stopped by heat treatment for 5 min at 95°C. Products were separated on 0.8% agarose gel. γ-PGA in the gel was visualized with methylene blue stain.

### Dynamic light scattering

Dynamic light scattering (DLS) measurements were performed using a DynaPro NanoStar instrument (Wyatt Technology Europe GmbH, Germany) with a 50-μl cuvette. The protein concentration used was about 10 mg/ml. All the DLS measurements were performed at 25°C and at an angle of 90°. The data were analyzed with the Dynamics v7.0 software.

## Results and discussion

### Models of the three PgdS NlpC/P60 domains

**Fig 1:**
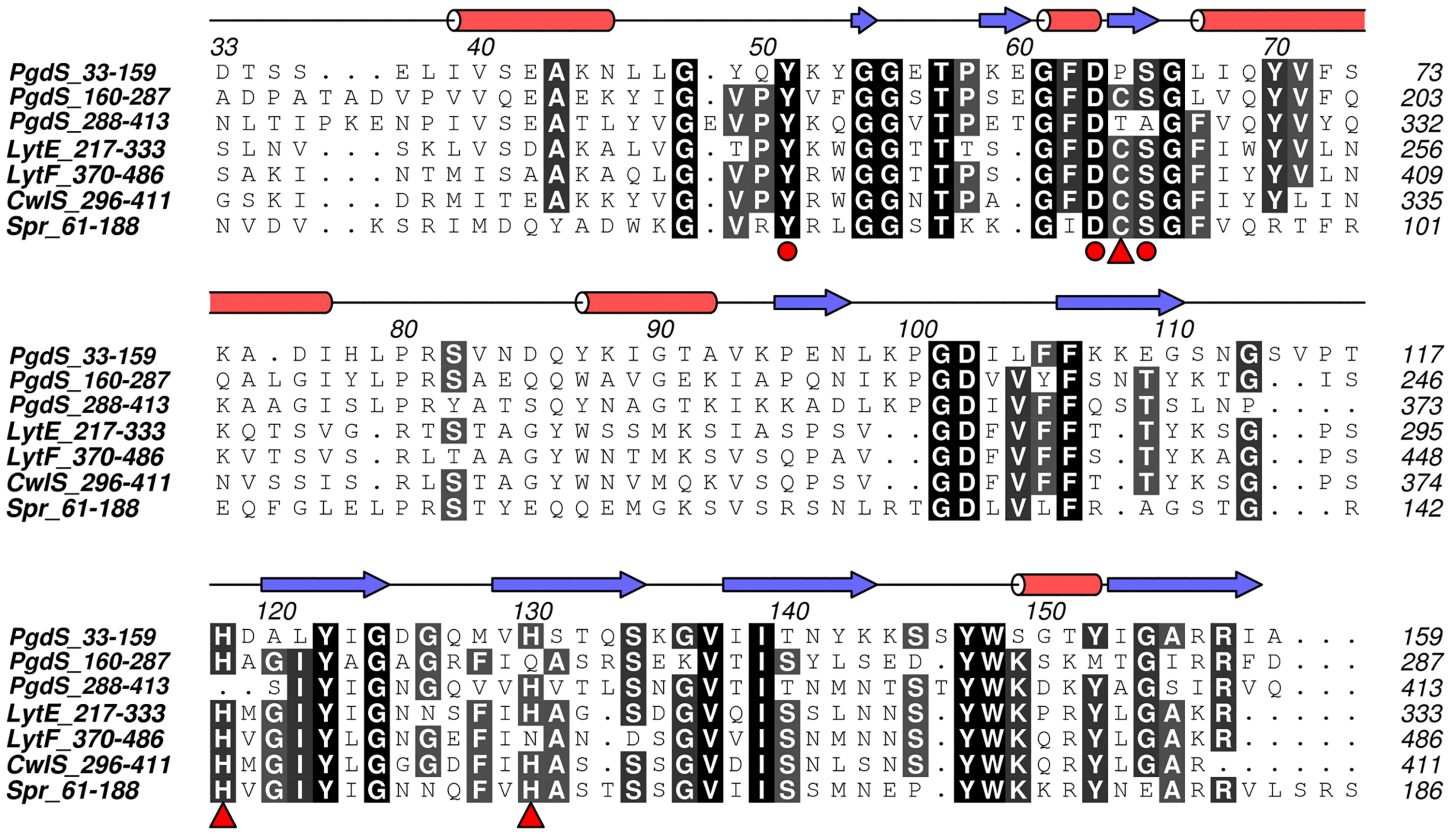
Multiple sequence alignment of three domains of PgdS and other NlpC/P60 domains. Three domains of PgdS, the NlpC/P60 catalytic domains of LytF, LytE and CwlS from *B*. *subtilis*, and putative lipoprotein Spr from *E*. *coli* (UniProt identifiers, P96740, P54421, O07532, O31852 and P0AFV4) are aligned with MUSCLE [27] and edited by hand to match the structural similarity where appropriate by using ALINE [28]. Identical and similar residues are highlighted in black and grey, respectivey. The secondary structure elements base on the domain 2 of PgdS, α-helices and β-strands are marked by red pillar and blue arrow, respectively. The strictly conserved cysteine/histidine/glutamine (asparagine or histidine) catalytic triad are marked with red triangles. Three conserved residues that contribute to the formation of catalytic core are also marked with red circles.

**Fig 2:**
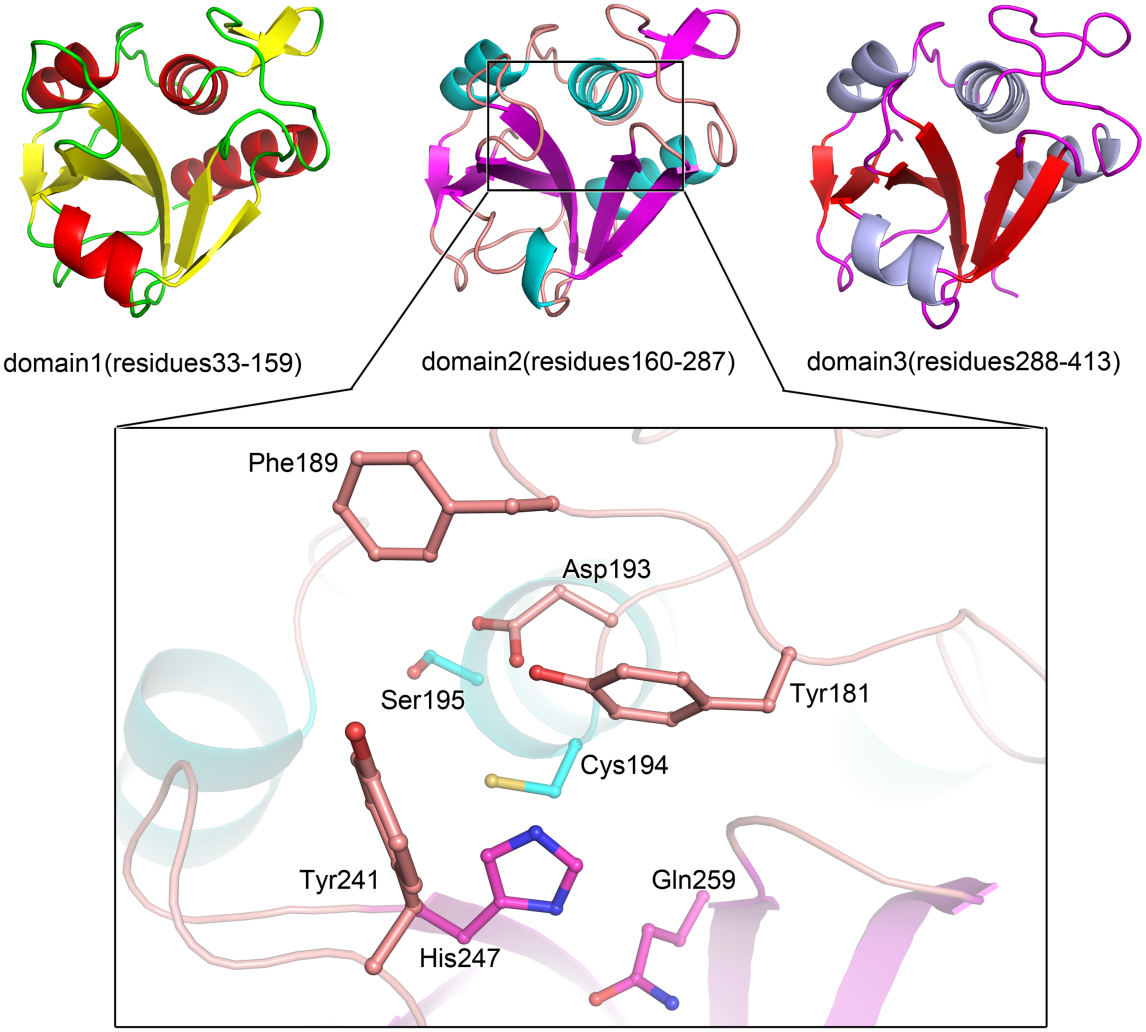
Overall fold of three domains of PgdS from *B*. *subtilis* as predicted using secondary and tertiary structure modeling. The α-helices and β-strands of three domains are colored with different colors (upper). The catalytic core of domain 2 is showed in the lower panel.

To date, the high resolution X-ray crystal structure of PgdS from *B*. *subtilis* has not been determined. We have tried a structure determination of the PgdS enzyme, but we failed. PgdS belongs to NlpC/P60 family, and is characterized as DL-endopeptidases [12]. Sequences analyses reveal that three tandem repeats of the NlpC/P60 module present in the protein PgdS, each of them has about 35% identity to the sequence of NlpC/P60 family [12] (Fig 1). So we compromised and used a combination of well-established secondary and tertiary structure modeling programs to develop an atomistic representation of the three PgdS domains. The prediction of the secondary structure of PgdS shows alternating pattern between α-helices and β-strands along the length of sequence in common with three tandem repeats of the NlpC/P60 fold (S1 Fig). Further, the tertiary structure of the three domains of PgdS were modeled using SWISS-MODEL [24]. The N-terminal domain 1 (residues 33–159) was predicted based on the structure of the NlpC/P60 domain in a putative cell wall hydrolase Tn916-like protein (PDB: 4HPE), which has a 37.5% sequence identity to PgdS, whereas the middle domain 2 (residues 160–287) and the C-terminal domain 3 (residues 288–413) were both predicted on the structure of a lipoprotein (PDB: 4FDY), with a sequence identity of 42.2% and 39.3%, respectively. All the three models present a typical NlpC/P60 fold, which is made up of a central β-sheet composed of five antiparallel β-strands that are surrounded by four α-helices, and the three models are extremely similar with a root-mean-square deviation (r.m.s.d) value ranging from 0.3–0.7 Å over all Cα atoms. Only small differences are occurred in the length of secondary structures and in the loop that links them (Fig 2). The geometry of the models was further validated by Procheck [25] and WhatIf [26]. The resulting Ramachandran reveals that over 80% of the amino acids fall in the preferred *ϕ/ψ* peptide bond angle regions and the models contain only 2% outliers in disallowed regions. The overall geometry and packing validation parameters calculated by WhatIf [26] correspond to a good-quality model.

The NlpC/P60 domain is responsible for the catalytic activity, and is highly modular. Many NlpC/P60 proteins are usually characterized by a single catalytic NlpC/P60 domain, and associated with other components, such as LysM, SH3 and choline-binding domains, to form a multifunctional protein [12,29]. For instance, the *B*. *subtilis* autolysins LytF, LytE, and CwlS each with a multiple tandem repeat of the LysM and a single NlpC/P60 domains [29], are localized at cell-separation sites during vegetative growth [30,31]. To date, several structures of the NlpC/P60 proteins have been solved with their fused domains and the single catalytic NlpC/P60 domains [32–36]. Beyond that, the RflaF_05439 from *Ruminococcus flavefaciens* is the only current example of a duplicated NlpC/P60 domain [37], and the PgdS from *B*. *subtilis* even carries three copies of this domain. In the PgdS enzyme, it has been demonstrated that only the second repeat of NlpC/P60 domain is functional [12,13], but no mention is made in the function of the other two repeats. In next, SAXS combining with other techniques are used to study the full length PgdS, which can give useful hints into the mechanisms of γ-PGA degradation by the PgdS hydrolase.

### Solution structure of PgdS at pH 6.0

**Fig 3:**
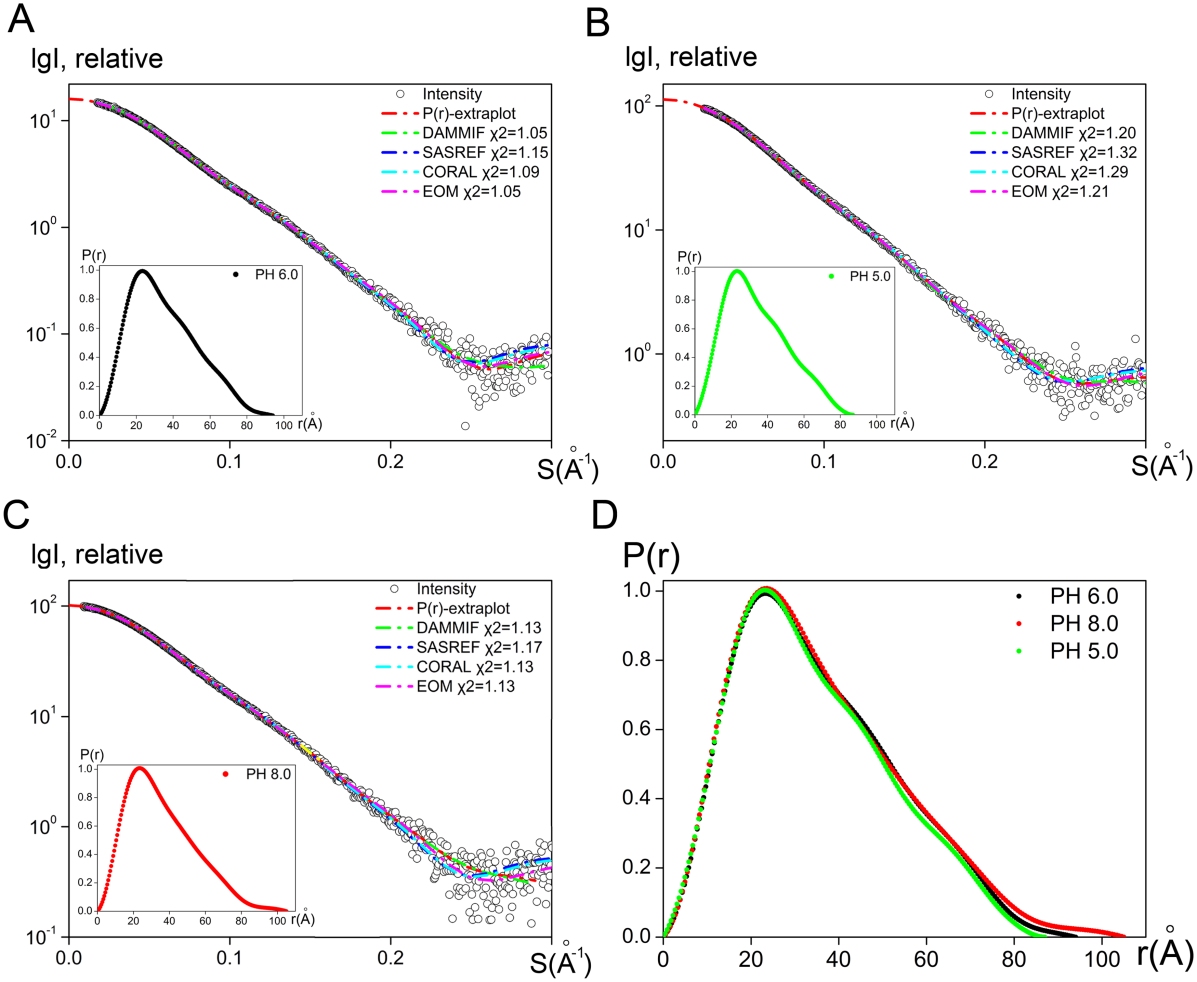
SAXS analyses of PgdS. SAXS scattering profiles and model reconstructions of PgdS at pH 6.0 (A), 5.0 (B) and 8.0 (C): black circle—experimental intensity; red line—smooth curve back transformed from the *p*(*r*) and extrapolated to zero scattering angle; green line—scattering pattern computed from the DAMMIF model; blue line—scattering pattern computed from the SASREF model; cyan line—scattering pattern computed from the CORAL model; magenta line—averaged scattering pattern calculated from the optimized models generated by EOM; lower left panel—distance distribution function *p(r)* for PgdS in solution. (D) normalized distance distribution functions for PgdS at pH 5.0 (black), pH 6.0 (green) and pH 8.0 (red).

Initially, we collected SAXS data for the full length PgdS in the purification buffer of 50 mM MES (pH 6.0) and 100 mM NaCl. No concentration dependence and aggregations were observed during the SAXS measurements (S2 Fig). The SAXS profiles for the PgdS at pH 6.0 are shown in Fig 3A. Molecular mass (MM) of the PgdS calculated from SAXS data is practically identical to the theoretical value calculated from known sequences, indicating well behaved, monodisperse status of a monomeric state in solution (S1 Table), which is consistent with size-exclusion chromatography results (data not shown). The distance distribution function *p(r)* for PgdS is shown in Fig 3D. Profiles of *p(r)* function for PgdS in solution is characterized as elongated body with cross-sections of ~ 22 Å and maximal particle dimension *D*_*max*_ of ~ 93 Å. To obtain more specific structural information, *ab initio* modeling is applied using the program DAMMIF [18]. Twenty independent models generated with the algorithms give reproducible results and demonstrate good approximations to the experimental data with a discrepancy value χ2 = 1.05 for the PgdS (Fig 3A, green line). The final models display on elongated shape for PgdS, consistent with the *p(r)* function.

**Fig 4:**
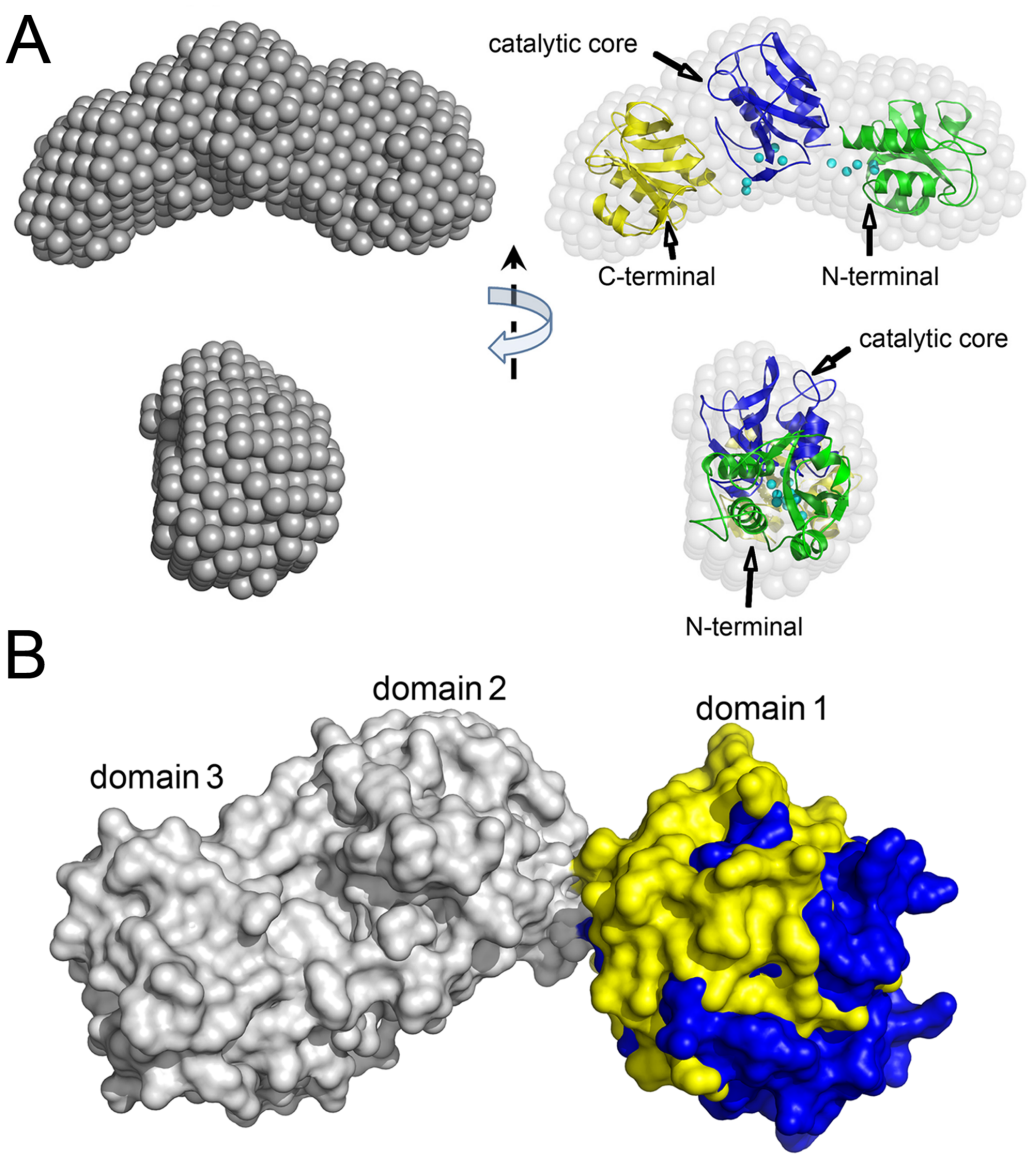
Model reconstructions of PgdS in solution. (A) Model reconstructions of PgdS in solution. Right: *ab initio* models from DAMMIF model; left, superposition of DAMMIF and CORAL model. The missing loops are represented as dummy residues colored cyan. Two orientations are shown. The domain 1, 2 and 3 are colored in green, blue and yellow, respectively. The relative positions of the N/C terminal and the functional catalytic core are also labeled. (B) Superimposed of two models of PgdS in a compact state (pH 5.0) and in an extended state (pH 8.0). The domain 2 and domain 3 are colored in grey, whereas domain 1 are colored in yellow (compact state) or blue (extended state).

A more detailed model of the full length structure of PgdS was generated using the three domains generated by SWISS-MODEL as a rigid body for SASREF [20] modeling. The rigid model of SASREF fits the experimental data very well (χ2 = 1.15) (Fig 3A, blue line). The SASREF model reveals the domains arrange as a crescent-shaped body with the domain 1 slightly apart from the other two. To further refine the rigid model, restorations of the linker loops between the domains were performed by CORAL [21] using SASREF model as a basis. Based on the PsiPred [23] results, two loop regions (residues 158–163, 288–294) connecting the three domains are defined. The results of the restorations by the program CORAL yield good fits to the experimental SAXS data (χ2 = 1.09) (Fig 3A, cyan line), too. Importantly, the CORAL reconstructions are in good agreement with the DAMMIF models as demonstrated in Fig 4A. Thus, two independent methods give consistent results, thereby supporting the notion that the models presented here clearly represent solution structures.

### The pH effect on the conformation of PgdS

The solution structure of PgdS was also investigated at pH 5.0 and pH 8.0, similar to the PgdS at pH 6.0 (S2 Fig). The scattering patterns and the *p(r)* functions of PgdS are shown in Fig 3B–3D. The estimated molecular mass of both also suggest a monomeric state for PgdS in solution that are consistent with the expected value (S1 Table). The real-space of *R*_*g*_ and *D*_*max*_ of PgdS at pH 5.0 are decreased noticeable as compared with those of the protein at pH 8.0, with *R*_*g*_ of 25.8 Å and *D*_*max*_ of ~87 Å for pH 5.0 and *R*_*g*_ of ~27.6 Å and *D*_*max*_ of ~27.6 Å for pH 8.0 (S1 Table). The decreasing in the both values of *R*_*g*_ and *D*_*max*_ indicate a more compact status of the PgdS at pH 5.0 compared to that of at pH 8.0. To further confirm the obtained results, dynamic light scattering analyses measurements were performed. The hydrodynamic radius *R*_*h*_ of PgdS at pH 8.0 was 3.0 nm, whereas the corresponding *R*_*h*_ of PgdS at pH 5.0 and 6.0 are both 2.8 nm (S3 Fig). The *R*_*h*_ values from DLS are nearly in line with the *R*_*g*_ values from SAXS, which demonstrate a similar structural conformation of PgdS at different pH values. We next performed *ab initio* shape reconstructions and rigid body refinement on the PgdS at pH 5.0 and pH 8.0, similar to the strategy described above for the PgdS at pH 6.0. Models generated by DAMMIF [18], SASREF [20] and CORAL [21] are in good agreement as show in Fig 3B and 3C. Both the models of the PgdS proteins at pH 5.0 and pH 8.0 exhibit a crescent-shaped bodies, with the domain 2 and domain 3 coordinated tightly, similar to the reconstruction model of PgdS at pH 6.0. However, the N-terminal domain 1 in PgdS at pH 5.0 arranges closer to the other two domains than that in PgdS at pH 8.0, which may be cause of the decreasing in *R*_*g*_ and *D*_*max*_ (Fig 4B).

**Fig 5:**
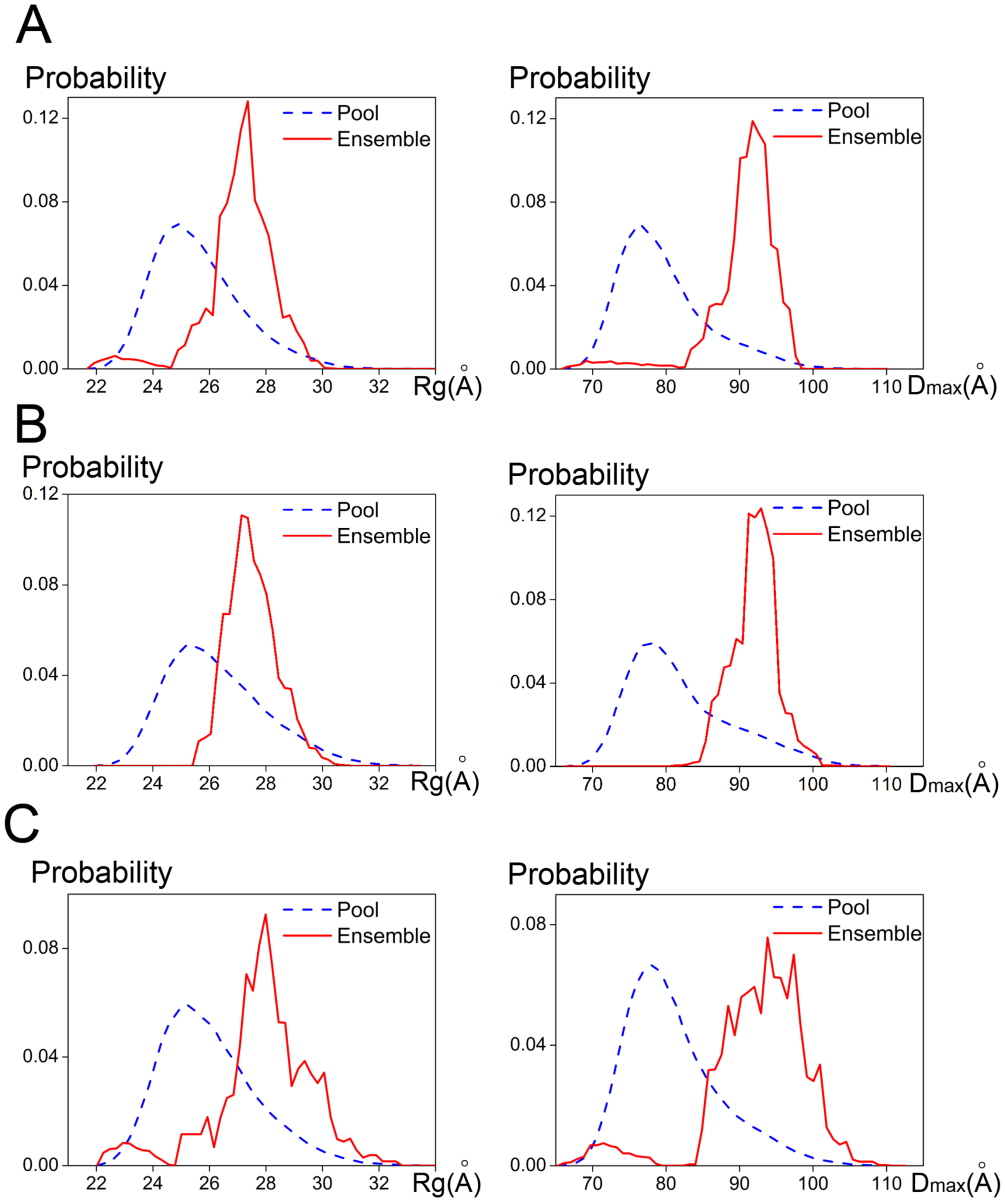
*R*_*g*_ and *D*_*max*_ distributions of the optimized ensembles for PgdS at various pH analyzed by program EOM. (A), (B) and (C) represent the distributions of *R*_*g*_ (*left*) and *D*_*max*_ (*right*) for PgdS at pH 5.0, pH 6.0 and pH 8.0, respectively.

To further validate the assumptions, EOM [22] was used to describe the PgdS proteins. Using the program EOM, a large pool of 10,000 different conformations is generated to analyze the flexibility of the protein, and an optimized ensemble of 50 models that best describes the SAXS data is selected. For PgdS at pH 5.0 and pH 6.0, both the *R*_*g*_ and *D*_max_ distribution functions have a single peak with *R*_*g*_ around 27 Å and *D*_*max*_ around 92 Å, respectively (Fig 5A and 5B), which is basically consistent with the overall structural parameters from SAXS data. This implies PgdS at pH 5.0 and pH 6.0 may mainly exist in a compact state. However, for PgdS at pH 8.0, both the *R*_*g*_ and *D*_max_ distribution functions has a broaden peak, ranging from ~25 to 31 Å and ~88 to 107 Å, respectively (Fig 5C). This means the full-length protein has a degree of a flexibility at pH 8.0, which probably undergo continuous conformational changes in solution. Considering the optimal pH value of PgdS enzyme is 5.0, it seems that the compact state of PgdS may facilitates the catalytic reaction. Therefore, our results indicate that the PgdS becomes an extended state with the increasing of pH value, which is probably due to the N-terminal domain 1 extending from the other two domains. In contrast, the domain 2 and 3 coordinate rigidly with limited flexibility, regardless the environment pH.

### Catalytic core and possible substrate delivery route

PgdS protein carries three copies of the NlpC/P60 domain, of which only the second is functional [13]. The NlpC/P60 domain represents a family of papain-like cysteine peptidases with a strictly conserved cysteine/histidine/glutamine (asparagine or histidine) catalytic triad [12]. Indeed, the multiple sequence alignment analysis of PgdS reveals that only the domain 2 has the complete catalytic triad, the Cys194-His247-Gln259, whereas the domain 1 and 3 have residue proline or threonine instead of cysteine, respectively (Fig 1). Overall, from the CORAL models of PgdS, the two core of domain 1 and domain 3 lie along two sides of the full length models of PgdS, whereas the functional catalytic core of domain 2 is next to the interface of domain 2 and domain 3, and resides in the outside of the crescent-shaped body, in the opposite side of the N/C-terminal of the full length PgdS (Fig 3A). The domain 2 represents a typical NlpC/P60 catalytic domain with a strictly conserved catalytic core (Fig 1). Around the catalytic cysteine, an aspartate, a serine and a tyrosine are strictly conserved in the domain 2 of PgdS (corresponding residues Asp193, Ser195 and Tyr181), which are presumed to relate to the substrate binding specifically. Besides, the conserved phenylalanine and tyrosine are also exist in the domain 2 of PgdS (corresponding residue Phe183 and Tyr241). Recent studies suggest these residues likely contribute as the gate accessing to catalytic core, which the side-chain of the phenylalanine can switches to a different rotamer to expose the catalytic core for substrate binding or product release [34].

**Fig 6:**
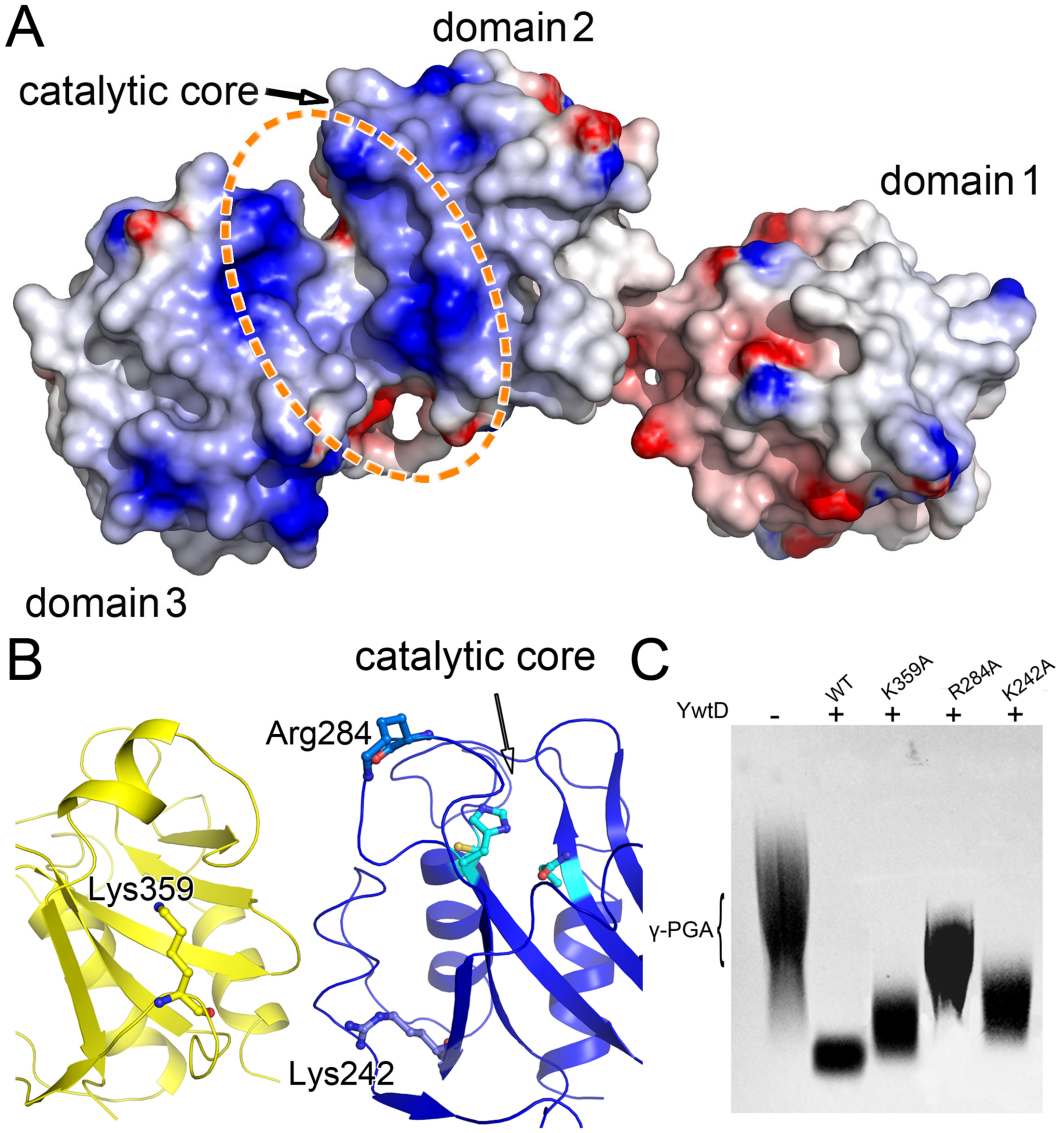
Possible substrate delivery route. (A) Electrostatic potential properties of PgdS, which are contoured over the range ± 5 kT/e using DelPhi [38] within the PyMOL (http://www.pymol.sourceforge.net/) software (blue represents a positively charged surface region and red represents a negatively charged surface region). The green circles signify the interface between domain 2 and domain 3. (B) The mutation sites in the positively charged surface at the junction of domain 2 and domain 3. (C) Activity of PgdS wild type and mutants.

The electronic surface of PgdS obtained from the CORAL models are presented in Fig 6A. A ~20 Å positively charged surface is localized on PgdS at the junction of domain 2 and domain 3 This positively charged surface runs along the interface from the inside to outside of the crescent-shaped body and extended to the catalytic core of the domain 2 through the gate of the Phe183 and Tyr241 [34]. Several basic amino acid from the two domains, like as Lys359, Arg284, Lys223 and Lys242 *et*.*al* reside in this region (Fig 6B). To investigating the possible function of the positively charged surface, three residues Lys359, Arg284 and Lys242 are mutated. Interesting, all the three PgdS mutants displayed defects in their ability to degrade the γ-PGA compared with the wild-type enzyme (Fig 6C). In an 2 hours reaction, the mutant K359A and K242A have lower efficiency reduction on γ-PGA degradation, in contrast, the mutant R284A has obvious decreasing in γ-PGA degradation. The result suggest that these residues are involved in the catalytic reaction, although all of them are far away from the catalytic core. PgdS is characterized as DL-endopeptidases, which exclusively cleaves the γ-glutamyl bond between D- and L-glutamic acids [13]. In this context, the way the enzyme distinguishes the compatible γ-glutamyl bonds in the long polymer of γ-PGA is very likely based on the cooperation of the domains, therefore, this long positively charged area between the domain 2 and domain 3 may server as a substrate delivery route between the enzyme and the γ-PGA.

A positively charged surface was also observed around the active site of other NlpC/P60 fused proteins, such as the *B*. *subtilis* autolysins DL-endopeptidases LytF, LytE, CwlS [39], and the poly-γ-glutamate hydrolase P (PghP) from bacteriophage ФNIT1 [40]. Moreover, it has been demonstrated that an inhibitor protein IseA, can get stuck deep in the cleft of LytF and occlude the active site by the interaction of the positively charged surface [39]. It must be noted that, there is still a limited understanding of how the enzyme anchor onto γ-PGA and how the substrates are delivered to the catalytic domain, as the mechanism of substrate delivery and recognition by PgdS is not firmly established in this study.

## Conclusion

In summary, the study presented here give the first depiction of the full-length PgdS protein. Although being a low-resolution method, SAXS can provide useful overall structural information of PgdS, such as the crescent-shaped body of the full length protein, the positively charged surface at the interface of domain 2 and 3, which may be relevant to its biological function. In order to fully understanding the mechanisms of γ-PGA degradation by the PgdS hydrolase, the high resolution structure of PgdS is still wanting. In addition, the results in this study will also provide valuable practical information for a controllable degradation of γ-PGA by enzymatic depolymerization.

## Supporting information

S1 Fig Predicted secondary structure of full length PgdS using PsiPred server. α-helices are shown as cylinders, β-strands as arrows and coils as a thick line. The confidence of the secondary structure prediction is plotted under each amino acid of the primary sequence.
(TIF)

S2 Fig Experimental SAXS patterns of PgdS at various pH. (A) pH 5.0, (B) pH6.0, (C) pH 8.0. The concentrations of PgdS are in the range of 1.1 to 7.2 mg/ml. No concentration dependence and aggregations were observed during the measurements.
(TIF)

S3 Fig Dynamic light scattering analyses of PgdS protein. Comparison of *R*_*h*_ values of PgdS by using DLS methods obtained at pH 5.0 (black), 6.0 (red) and 8.0 (blue).
(TIF)

S1 Table Overall structural parameters of the PgdS proteins at various pH from SAXS data. (DOC)

## References

[1] Shih IL, Van YT (2001) The production of poly-(gamma-glutamic acid) from microorganisms and its various applications. Bioresour Technol 79: 207–225. 11499575 10.1016/s0960-8524(01)00074-8

[2] Yu YY, Yan F, Chen Y, Jin C, Guo JH, Chai HR. (2016) Poly-gamma-Glutamic Acids Contribute to Biofilm Formation and Plant Root Colonization in Selected Environmental Isolates of Bacillus subtilis. Front Microbiol 7. 10.3389/fmicb.2016.01811 PMC5102903 27891125

[3] Bhat AR, Irorere VU, Bartlett T, Hill D, Kedia G, Morris MR, et al (2013) Bacillus subtilis natto: a non-toxic source of poly-gamma-glutamic acid that could be used as a cryoprotectant for probiotic bacteria. AMB Express 3: 36 doi: 10.1186/2191-0855-3-36 23829836 10.1186/2191-0855-3-36 PMC3720193

[4] Peng YY, Jiang B, Zhang T, Mu WM, Miao M, Hua YF. (2015) High-level production of poly(gamma-glutamic acid) by a newly isolated glutamate-independent strain, Bacillus methylotrophicus. Process Biochem 50: 329–335.

[5] Bajaj I, Singhal R (2011) Poly (glutamic acid)—an emerging biopolymer of commercial interest. Bioresour Technol 102: 5551–5561. doi: 10.1016/j.biortech.2011.02.047 21377358 10.1016/j.biortech.2011.02.047

[6] Yan S, Yao HS, Chen Z, Zeng SQ, Xi X, Wang YP, et al (2015) Poly-gamma-glutamic acid produced from Bacillus licheniformis CGMCC 2876 as a potential substitute for polyacrylamide in the sugarcane industry. Biotechnol Progr 31: 1287–1294. 10.1002/btpr.2118 26033934

[7] Tanimoto H, Fox T, Eagles J, Satoh H, Nozawa H, Okiyama A, et al (2007) Acute effect of poly-gamma-glutamic acid on calcium absorption in post-menopausal women. J Am Coll Nutr 26: 645–649. 18187428 10.1080/07315724.2007.10719642

[8] Zhao C, Zhang Y, Wei X, Hu Z, Zhu F, Xu L, et al (2013) Production of ultra-high molecular weight poly-gamma-glutamic acid with Bacillus licheniformis P-104 and characterization of its flocculation properties. Appl Biochem Biotechnol 170: 562–572. doi: 10.1007/s12010-013-0214-2 23553109 10.1007/s12010-013-0214-2

[9] Yao J, Jing J, Xu H, Liang JF, Wu Q, Feng XH, et al (2009) Investigation on enzymatic degradation of gamma-polyglutamic acid from Bacillus subtilis NX-2. J Mol Catal B-Enzym 56: 158–164.

[10] Huang J, Du YM, Xu GH, Zhang HL, Zhu F, Huang L, et al (2011) High yield and cost-effective production of poly(gamma-glutamic acid) with Bacillus subtilis. Eng Life Sci 11: 291–297.

[11] Cao M, Geng W, Liu L, Song C, Xie H, Guo W, et al (2011) Glutamic acid independent production of poly-gamma-glutamic acid by Bacillus amyloliquefaciens LL3 and cloning of pgsBCA genes. Bioresour Technol 102: 4251–4257. doi: 10.1016/j.biortech.2010.12.065 21232939 10.1016/j.biortech.2010.12.065

[12] Anantharaman V, Aravind L (2003) Evolutionary history, structural features and biochemical diversity of the NlpC/P60 superfamily of enzymes. Genome Biol 4: R11 doi: 10.1186/gb-2003-4-2-r11 12620121 10.1186/gb-2003-4-2-r11 PMC151301

[13] Suzuki T, Tahara Y (2003) Characterization of the Bacillus subtilis ywtD gene, whose product is involved in gamma-polyglutamic acid degradation. J Bacteriol 185: 2379–2382. doi: 10.1128/JB.185.7.2379-2382.2003 12644511 10.1128/JB.185.7.2379-2382.2003 PMC151509

[14] Petersen TN, Brunak S, von Heijne G, Nielsen H (2011) SignalP 4.0: discriminating signal peptides from transmembrane regions. Nat Methods 8: 785–786. doi: 10.1038/nmeth.1701 21959131 10.1038/nmeth.1701

[15] Li N, Li XH, Wang YZ, Liu GF, Zhou P, Wu HJ, et al (2016) The new NCPSS BL19U2 beamline at the SSRF for small-angle X-ray scattering from biological macromolecules in solution. J Appl Crystallogr 49: 1428–1432. doi: 10.1107/S160057671601195X 27738413 10.1107/S160057671601195X PMC5045727

[16] Franke D, Petoukhov MV, Konarev PV, Panjkovich A, Tuukkanen A, Mertens HDT, et al (2017) ATSAS 2.8: a comprehensive data analysis suite for small-angle scattering from macromolecular solutions. J Appl Crystallogr 50: 1212–1225. doi: 10.1107/S1600576717007786 28808438 10.1107/S1600576717007786 PMC5541357

[17] Konarev PV, Volkov VV, Sokolova AV, Koch MHJ, Svergun DI (2003) PRIMUS: a Windows PC-based system for small-angle scattering data analysis. J Appl Crystallogr 36: 1277–1282.

[18] Franke D, Svergun DI (2009) DAMMIF, a program for rapid ab-initio shape determination in small-angle scattering. J Appl Crystallogr 42: 342–346. doi: 10.1107/S0021889809000338 27630371 10.1107/S0021889809000338 PMC5023043

[19] Volkov VV, Svergun DI (2003) Uniqueness of ab initio shape determination in small-angle scattering. J Appl Crystallogr 36: 860–864. 10.1107/S0021889809000338 PMC5023043 27630371

[20] Petoukhov MV, Svergun DI (2005) Global rigid body modeling of macromolecular complexes against small-angle scattering data. Biophys J 89: 1237–1250. doi: 10.1529/biophysj.105.064154 15923225 10.1529/biophysj.105.064154 PMC1366608

[21] Petoukhov MV, Franke D, Shkumatov AV, Tria G, Kikhney AG, Gajda M, et al (2012) New developments in the ATSAS program package for small-angle scattering data analysis. J Appl Crystallogr 45: 342–350. doi: 10.1107/S0021889812007662 25484842 10.1107/S0021889812007662 PMC4233345

[22] Tria G, Mertens HD, Kachala M, Svergun DI (2015) Advanced ensemble modelling of flexible macromolecules using X-ray solution scattering. IUCrJ 2: 207–217. doi: 10.1107/S205225251500202X 25866658 10.1107/S205225251500202X PMC4392415

[23] Buchan DW, Minneci F, Nugent TC, Bryson K, Jones DT (2013) Scalable web services for the PSIPRED Protein Analysis Workbench. Nucleic Acids Res 41: W349–357. doi: 10.1093/nar/gkt381 23748958 10.1093/nar/gkt381 PMC3692098

[24] Biasini M, Bienert S, Waterhouse A, Arnold K, Studer G, Schmidt T, et al (2014) SWISS-MODEL: modelling protein tertiary and quaternary structure using evolutionary information. Nucleic Acids Res 42: W252–258. doi: 10.1093/nar/gku340 24782522 10.1093/nar/gku340 PMC4086089

[25] Laskowski RA, Macarthur MW, Moss DS, Thornton JM (1993) Procheck—a Program To Check the Stereochemical Quality Of Protein Structures. J Appl Crystallogr 26: 283–291.

[26] Vriend G (1990) What If—a Molecular Modeling And Drug Design Program. J Mol Graphics 8: 52-&. 10.1016/0263-7855(90)80070-v 2268628

[27] Edgar RC (2004) MUSCLE: multiple sequence alignment with high accuracy and high throughput. Nucleic Acids Res 32: 1792–1797. doi: 10.1093/nar/gkh340 15034147 10.1093/nar/gkh340 PMC390337

[28] Bond CS, Schuttelkopf AW (2009) ALINE: a WYSIWYG protein-sequence alignment editor for publication-quality alignments. Acta Crystallogr D Biol Crystallogr 65: 510–512. doi: 10.1107/S0907444909007835 19390156 10.1107/S0907444909007835

[29] Smith TJ, Blackman SA, Foster SJ (2000) Autolysins of Bacillus subtilis: multiple enzymes with multiple functions. Microbiology 146 (Pt 2): 249–262. 10708363 10.1099/00221287-146-2-249

[30] Yamamoto H, Kurosawa S, Sekiguchi J (2003) Localization of the vegetative cell wall hydrolases LytC, LytE, and LytF on the Bacillus subtilis cell surface and stability of these enzymes to cell wall-bound or extracellular proteases. J Bacteriol 185: 6666–6677. doi: 10.1128/JB.185.22.6666-6677.2003 14594841 10.1128/JB.185.22.6666-6677.2003 PMC262103

[31] Fukushima T, Afkham A, Kurosawa S, Tanabe T, Yamamoto H, Sekiguchi J. (2006) A new D,L-endopeptidase gene product, YojL (renamed CwlS), plays a role in cell separation with LytE and LytF in Bacillus subtilis. J Bacteriol 188: 5541–5550. doi: 10.1128/JB.00188-06 16855244 10.1128/JB.00188-06 PMC1540035

[32] Aramini JM, Rossi P, Huang YJ, Zhao L, Jiang M, Maglaqui M, et al (2008) Solution NMR structure of the NlpC/P60 domain of lipoprotein Spr from Escherichia coli: structural evidence for a novel cysteine peptidase catalytic triad. Biochemistry 47: 9715–9717. doi: 10.1021/bi8010779 18715016 10.1021/bi8010779

[33] Wong JE, Midtgaard SR, Gysel K, Thygesen MB, Sorensen KK, Jensen KJ, et al (2015) An intermolecular binding mechanism involving multiple LysM domains mediates carbohydrate recognition by an endopeptidase. Acta Crystallogr D Biol Crystallogr 71: 592–605. doi: 10.1107/S139900471402793X 25760608 10.1107/S139900471402793X PMC4356369

[34] Xu QP, Chiu HJ, Farr CL, Jaroszewski L, Knuth MW, Miller MD, et al (2014) Structures of a Bifunctional Cell Wall Hydrolase CwlT Containing a Novel Bacterial Lysozyme and an NlpC/P60 DL-Endopeptidase. J Mol Biol 426: 169–184. doi: 10.1016/j.jmb.2013.09.011 24051416 10.1016/j.jmb.2013.09.011 PMC3872209

[35] Xu Q, Sudek S, McMullan D, Miller MD, Geierstanger B, Jones DH, et al (2009) Structural basis of murein peptide specificity of a gamma-D-glutamyl-l-diamino acid endopeptidase. Structure 17: 303–313. doi: 10.1016/j.str.2008.12.008 19217401 10.1016/j.str.2008.12.008 PMC2667786

[36] Xu Q, Abdubek P, Astakhova T, Axelrod HL, Bakolitsa C, Cai X, et al (2010) Structure of the gamma-D-glutamyl-L-diamino acid endopeptidase YkfC from Bacillus cereus in complex with L-Ala-gamma-D-Glu: insights into substrate recognition by NlpC/P60 cysteine peptidases. Acta Crystallogr Sect F Struct Biol Cryst Commun 66: 1354–1364. doi: 10.1107/S1744309110021214 20944232 10.1107/S1744309110021214 PMC2954226

[37] Levy-Assaraf M, Voronov-Goldman M, Rozman Grinberg I, Weiserman G, Shimon LJ, Jindou S, et al (2013) Crystal structure of an uncommon cellulosome-related protein module from Ruminococcus flavefaciens that resembles papain-like cysteine peptidases. PLoS One 8: e56138 doi: 10.1371/journal.pone.0056138 23457513 10.1371/journal.pone.0056138 PMC3573020

[38] Li L, Li C, Sarkar S, Zhang J, Witham S, Zhang Z, et al (2012) DelPhi: a comprehensive suite for DelPhi software and associated resources. BMC Biophys 5: 9 doi: 10.1186/2046-1682-5-9 22583952 10.1186/2046-1682-5-9 PMC3463482

[39] Arai R, Fukui S, Kobayashi N, Sekiguchi J (2012) Solution structure of IseA, an inhibitor protein of DL-endopeptidases from Bacillus subtilis, reveals a novel fold with a characteristic inhibitory loop. J Biol Chem 287: 44736–44748. doi: 10.1074/jbc.M112.414763 23091053 10.1074/jbc.M112.414763 PMC3531787

[40] Fujimoto Z, Kimura K (2012) Crystal structure of bacteriophage Phi NIT1 zinc peptidase PghP that hydrolyzes gamma-glutamyl linkage of bacterial poly-gamma-glutamate. Proteins 80: 722–732. doi: 10.1002/prot.23229 22105902 10.1002/prot.23229

