# Supplementary figures and images for: Solution scattering study of the Bacillus subtilis PgdS enzyme involved in poly-γ-glutamic acids degradation
Source: PLoS One. 2018 Apr 2;13(4):e0195355. doi: 10.1371/journal.pone.0195355 (PMC5880399; doi:10.1371/journal.pone.0195355)

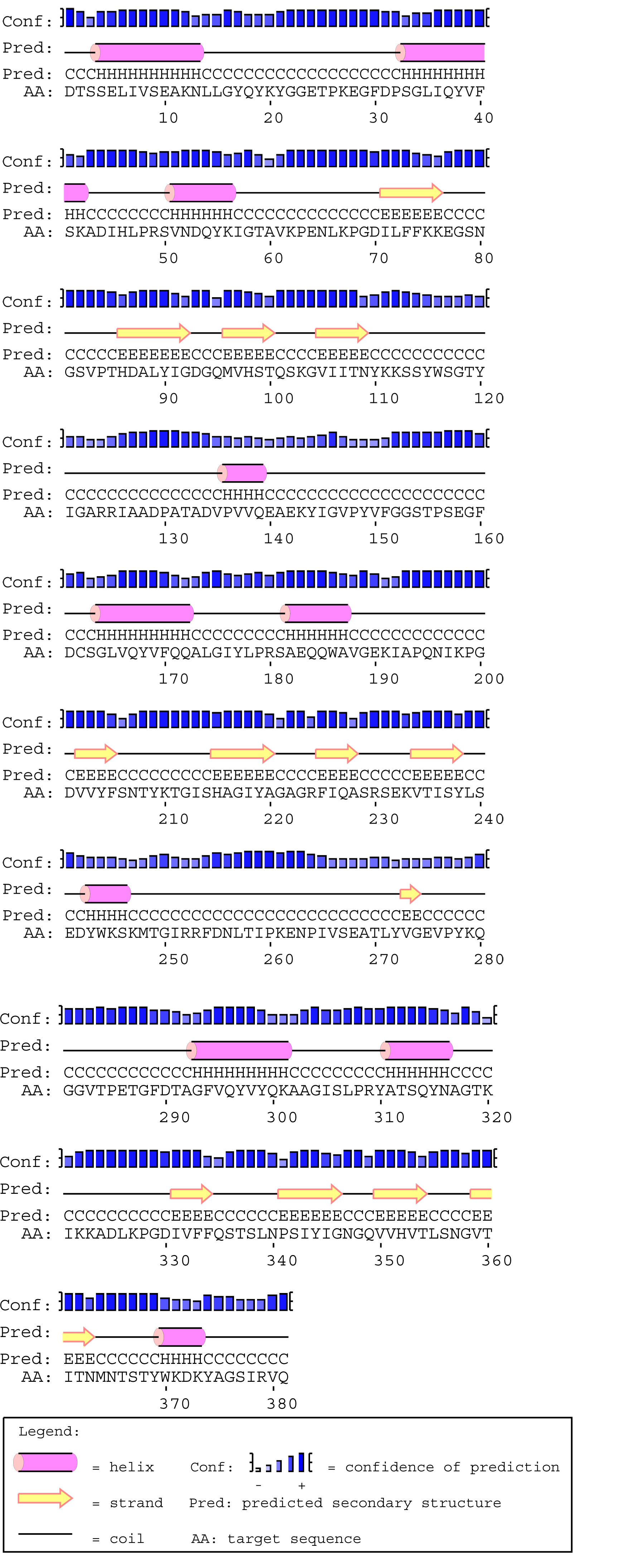

Supplement: S1 Fig — α-helices are shown as cylinders, β-strands as arrows and coils as a thick line. The confidence of the secondary structure prediction is plotted under each amino acid of the primary sequence. (TIF) [file pone.0195355.s001.tif]

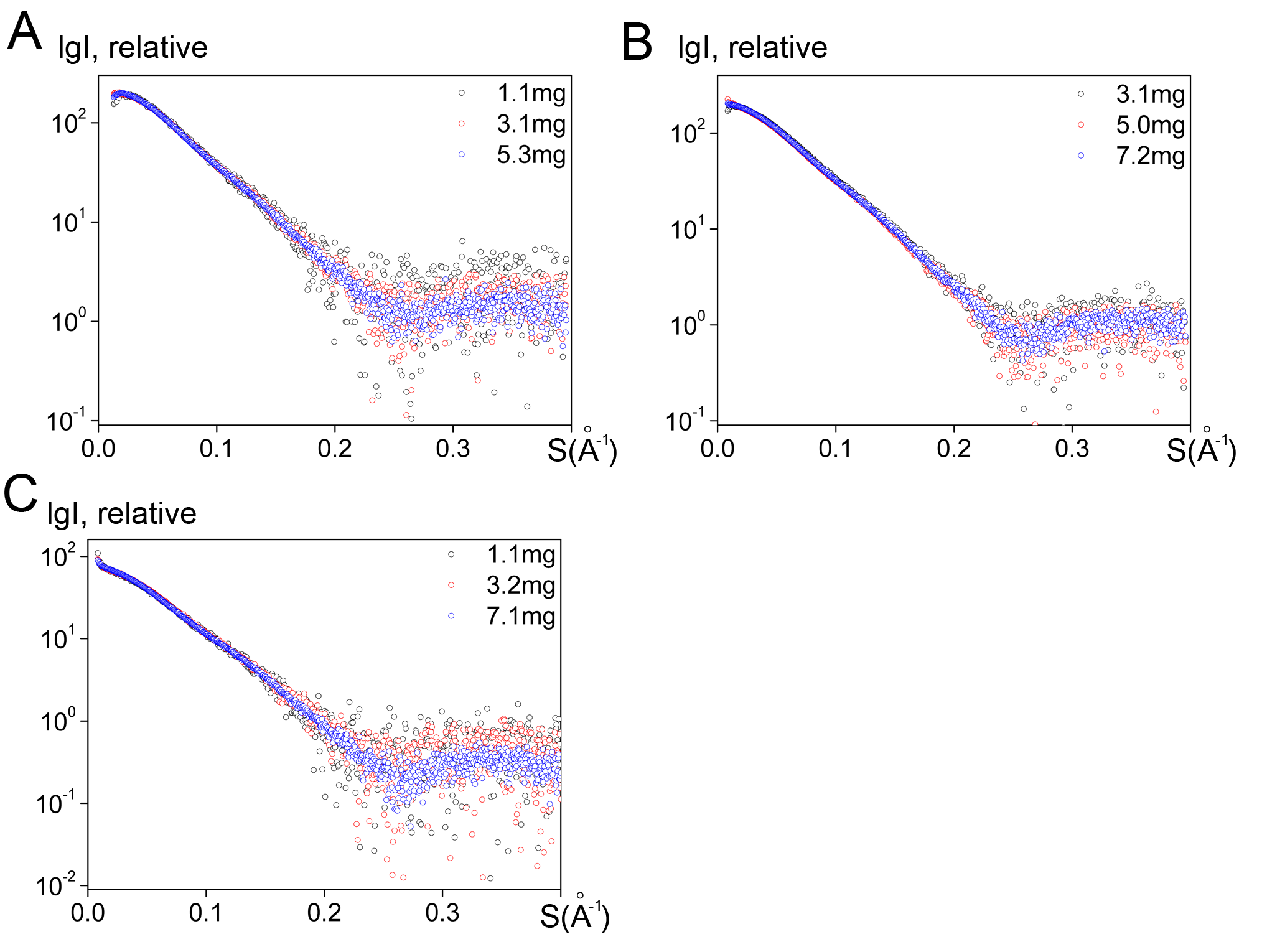

Supplement: S2 Fig — (A) pH 5.0, (B) pH6.0, (C) pH 8.0. The concentrations of PgdS are in the range of 1.1 to 7.2 mg/ml. No concentration dependence and aggregations were observed during the measurements. (TIF) [file pone.0195355.s002.tif]

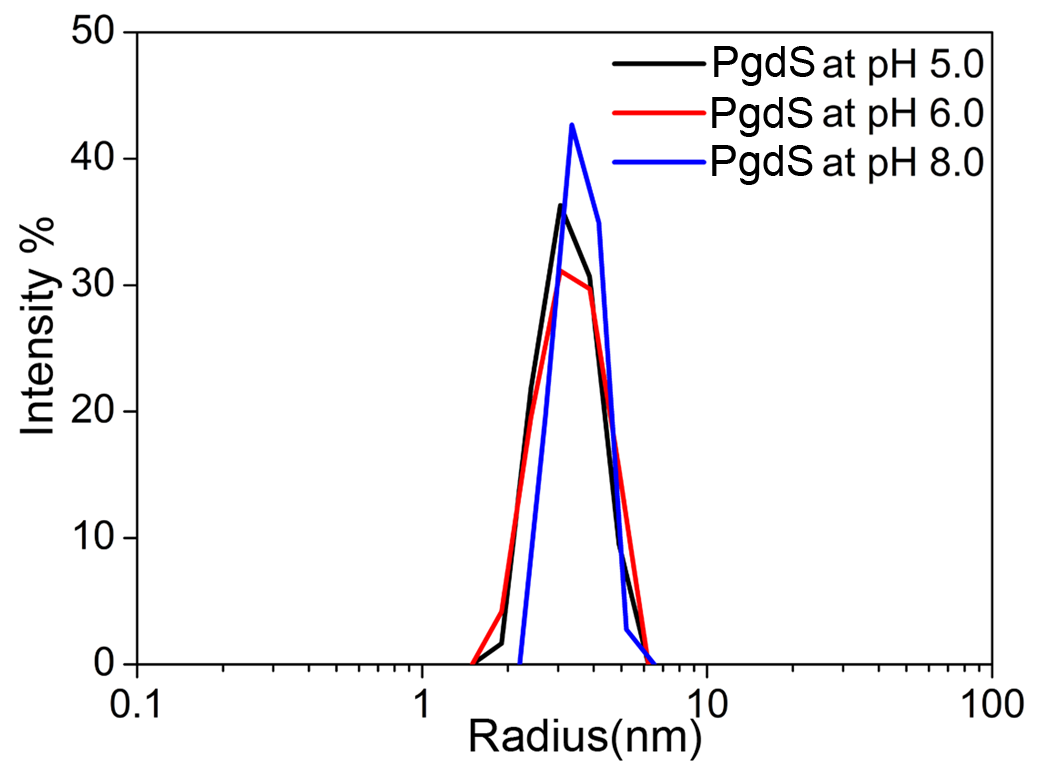

Supplement: S3 Fig — Comparison of Rh values of PgdS by using DLS methods obtained at pH 5.0 (black), 6.0 (red) and 8.0 (blue). (TIF) [file pone.0195355.s003.tif]
