# Supplementary material for: Solution scattering study of the Bacillus subtilis PgdS enzyme involved in poly-γ-glutamic acids degradation
Source: PLoS One. 2018 Apr 2;13(4):e0195355. doi: 10.1371/journal.pone.0195355 (PMC5880399; doi:10.1371/journal.pone.0195355)
Supplement: S1 Table — (DOC) [file pone.0195355.s004.doc]

**Solution scattering study of the *Bacillus subtilis* PgdS enzyme involved in poly-γ-glutamic acids degradation**

Jumei Zeng ^1^, Yun Jin^1^, Zhongchuan Liu^1*^

*^1^Key Laboratory of Environmental and Applied Microbiology, Chengdu Institute of Biology, Chinese Academy of Sciences, Chengdu, Sichuan, China;*

*Corresponding author:

*E-mail:* [*liuzhongch07@mails.ucas.ac.cn*](mailto:liuzhongch07@mails.ucas.ac.cn)

**S1 Table. Overall structural parameters of the PgdS proteins at various pH from SAXS data­^*^**

| Sample(PgdS) | *R_g_*, Å | *V_p_,* Å^3^ | *D_max_*, Å | *MM_I(0)_*, kDa | *MM_porod_*, kDa | *MM_sequence_*,kDa |
| --- | --- | --- | --- | --- | --- | --- |
| pH 5.0 | 25.8±0.07 | 68±4 | 87±3 | 40.1±2 | 42.5±3 | 41.7 |
| pH 6.0 | 26.8±0.08 | 64±3 | 93±3 | 40.7±2 | 41.2±2 | 41.7 |
| pH 8.0 | 27.6±0.05 | 66±4 | 107±5 | 41.3±2 | 42.0±2 | 41.7 |

^*^*R_g_*, radius of gyration from Guinier; *V_p_*, Porod volume estimate; *D_max_*, maximum particle dimension; *MM_I(0)_*, molecular mass from I(0); *MM_Porod_*, molecular mass from Porod volume; *MM_sequence_*, calculated oligomeric molecular mass from the amino acid.
